# Effectiveness of Hydroalcoholic Seed Extract of *Securigera securidaca* on Pancreatic Local Renin-Angiotensin System and Its Alternative Pathway in Streptozotocin-Induced Diabetic Animal Model

**DOI:** 10.1155/2023/7285036

**Published:** 2023-01-07

**Authors:** Fatemeh Khomari, Bahar Kiani, Shahin Alizadeh-Fanalou, Mohammad Babaei, Ali Kalantari-Hesari, Iraj Alipourfard, Fatemeh Mirzaei, Sahar Yarahmadi, Elham Bahreini

**Affiliations:** ^1^Department of Biochemistry, Faculty of Medicine, Iran University of Medical Sciences, Tehran, Iran; ^2^Nephrology and Kidney Transplant center, Clinical Research Institute, Urmia University of Medical Sciences, Urmia, Iran; ^3^Department of Clinical Sciences, Faculty of Veterinary Science, Bu-Ali Sina University, Hamedan, Iran; ^4^Institute of Biology, Biotechnology and Environmental Protection, Faculty of Natural Sciences, University of Silesia, Bankowa 9, 40-007 Katow, Poland; ^5^Department of Anatomical Sciences, School of Medicine, Hamedan University of Medical Sciences, Hamedan, Iran

## Abstract

**Background:**

Available data suggest inhibition of the pancreatic local-renin-angiotensin system (RAS) reduces tissue complications of diabetes. The purpose of the present study was to investigate the effect of hydroalcoholic seed extract of *Securigera securidaca* (*S. securidaca*) (HESS) on the pancreatic local-RAS and its alternative pathway.

**Methods:**

Three doses of HESS were orally administered to three groups of diabetic male Wistar rats, and the results were compared with both diabetic and healthy control groups. After 35 days of treatment, the groups were assessed for the levels of pancreatic local-RAS components, including renin, angiotensinogen, ACE, and Ang II, as well as ACE2 and Ang-(1-7) in the alternative pathway. The effect of herbal medicine treatment on tissue damage status was investigated by evaluating tissue levels of oxidative stress, proinflammatory and anti-inflammatory cytokines, and through histopathological examination of the pancreas.

**Results:**

HESS showed a dose-dependent palliative effect on the tissue oxidative stress profile (*P* < 0.05) as well as the levels of pancreatic local-RAS components (*P* < 0.05), compared to diabetic control group. Considering the interrelationship between tissue oxidative stress and local-RAS activity, the moderating effect of HESS on this relationship could be attributed to the increase in total tissue antioxidant capacity (TAC) and pancreatic Ang-(1-7) concentration. Decrease in local-RAS activity was associated with decrease in the tissue levels of inflammatory cytokines (IL1, IL6, and TNF*α*) (*P* < 0.05) and increase in the levels of anti-inflammatory cytokine of IL-10 (*P* < 0.05). In addition, histological results were consistent with tissue biochemical results.

**Conclusions:**

Due to the reduction of local pancreatic RAS activity as well as oxidative stress and proinflammatory cytokines following treatment with HESS, *S. securidaca* seed can be proposed as a suitable herbal supplement in the drug-treatment of diabetes.

## 1. Background

Insulin secretion is a process dependent on oxidative phosphorylation and oxygen consumption that triggers pancreatic *β*-cells to produce reactive oxygen/nitrogen species (RONS) [1]. As an active process, it is necessary to increase glycolysis and the tricarboxylic acid (TCA) cycle for proper insulin secretion. However, the high capacity of *β*-cells to transport glucose inward relative to the rate of TCA cycle and respiratory chain makes the high glucose concentrations a limiting factor for glycolysis [2, 3]. Sustained increases in glycolytic flux and insulin secretion may lead to the production of RONS in *β*-cells. The absence of an effective antioxidant system in *β*-cells makes them highly susceptible to oxidative stress, pancreatic hypoxia, and subsequent onset of diabetes [4, 5]. Oxidative stress and hypoxia activate the tissue components of the local renin-angiotensin system (RAS), which constrict pancreatic blood vessels and exacerbate inflammation and tissue damage [1].

In addition to circulating RAS, many tissues and organs, including the pancreas, liver, and heart, have their own local-RAS. Such local-RASs operate in an autocrine, paracrine, and/or intracrine manner and exhibit multiple physiological effects at the cellular level. Most studies have decleared that local tissue RAS components are independent of those found in the plasma [6, 7] and act locally independent from systemic RAS [8, 9]. The existence of a local-RAS has been recognized in different parts of pancreas. The expression of its components is regulated in response to physiological and pathophysiological stimuli such as pancreatitis, hypoxia, hyperglycemia, and diabetes [10].

The natural function of pancreatic local-RAS is to maintain homeostasis through islet survival and regulation of insulin secretion. In normal condition, the existence of local-RAS in pancreas plays an important role in the regulation of cell growth, differentiation, proliferation, and glucose-induced insulin secretion in islates [11]. In this system, the two major G protein-coupled receptors, angiotensin II type 1 (AT1R) and angiotensin II type 2 (AT2R), are mainly found in beta cells and neighboring delta cells, respectively [12, 13]. In this intrinsic system, angiotensinogen and angiotensin-converting enzyme (ACE) are mainly expressed in alpha cells and islet microvasculature, respectively [14]. Most of the physiological effects of Ang II are induced through AT1R receptor and are mediated by signaling molecules including protein kinases and small G-proteins (Ras, Rho, Rac, etc.) [15, 16].

In addition to haemodynamic actions, the local-RAS has multiple functions including apoptosis, reactive oxygen species (ROS) generation, tissue inflammation, and fibrosis. Therefore, dysfunction of this system associates with pathological changes in pancreatic and metabolic diseases. Upregulation of local-RAS components in the pancreas of diabetic and obese animals has been evidenced by several studies [17, 18]. Although RONS stimulates upregulation of pancreatic RAS, increased local-RAS activity also intensifies RONS production through numerous signaling pathways in response to Ang II [19]. Oxidative stress resulted inflammation inducing mitochondrial dysfunction through activation of uncoupling proteins 2 (UCP2). UCP2 acts as a sensor of mitochondrial oxidative stress, silences glucose oxidation in *β*-cells, and reduces mitochondrial function and insulin secretion in *β*-cells [20].

A reciprocal relationship exists between oxidative stress and the local-RAS system. Ang II, via Ang II, is a potent activator of NAD(P)H oxidase, which is the main driver of ROS production in various tissues. The proposed signaling pathway is the activation of G_*α*12/13_ proteins by Ang II, leading to Rho/ROCK-mediated activation of Rac1. Rac 1, a small GTP-binding protein, promotes ROS generation by activating NAD(P)H oxidase [21, 22].

The mild activity of local-RAS is important for tissue homeostasis, but hyperactivity is a devastating phenomenon observed in diabetic pancreas. Overactivity of the Ang II-AT1R impairs glucose metabolism due to oxidative stress and induction of inflammatory pathways, leading to the decreased activity of insulin receptor substrates (IRSs), phosphoinositide 3-kinase (PI3K), phospho-protein kinase B (AKT2), glucokinase (GCK), and glucose transporter 2 (GLUT2) [23]. However, an alternative system exists in tissues, that often counteract the deleterious effects of the ACE/Ang II/A AT1R axis in the local-RAS system. In this alternative system, angiotensin-converting enzyme 2 (ACE2), a homolog of ACE with 42% homology [24], is most commonly expressed in alpha cells and is involved in beneficial effects on glucose homeostasis and maintenance of the islets [25].

ACE2 catalyzes the conversion of Ang I to the nonapeptide Ang-(1–9) and the conversion of Ang-(1–9) and Ang II to the heptapeptide Ang-(1–7). The biological activity of Ang-(1–7) is mediated through the Mas receptor, leading to vasodilation and increased insulin secretion due to the release of bradykinin, nitric oxide [26], and prostaglandin [27, 28]. The ACE2/Ang-(1–7)/Mas axis influences insulin secretion at the posttranscriptional/posttranslational level. Sahr et al. suggested that Ang-(1-7) increases insulin secretion in *β*-cells by increasing intracellular cAMP via an increase in PKA or EPAC 2 [29].

The plant *Securigera securidaca* (L.) (bitter lentils) is a potent herb with hypoglycemic and hypolipidemic effects in traditional Iranian, Egyptian, and Indian medicine [30]. HPLC and GC-MS analysis of hydroalcoholic extract of *S. securidaca* seeds by Aldal'in et al. showed a rich content of flavonoids, saponins, tannins, and alkaloids in the seeds of this plant [31]. Their phytochemical analysis also showed high content of L-ascorbic acid, aromatic and dodecanedioic acid derivatives, and *β*-sitosterol and oxygenated hydrocarbons. In the previous studies, we demonstrated systemic properties of HESS, including antioxidant, anti-inflammatory, antidiabetic, and antihyperlipidemic effects [32–34]. The lack of previous studies on the effect of hydroalcoholic extract of *S. securidaca* (HESS) on pancreas tissue and its local-RAS fulfills the need to be investigated in this study.

## 2. Methods

### 2.1. Hydroalcoholic Extraction of Seed and Estimation of Total Phenolic and Flavonoid

The preparation of *S. securidaca* seed was performed according to IUCN policy (https://portals.iucn.org/library/efiles/documents/PP-003-En.pdf). *S. securidaca* seeds were purchased from a center for medicinal plants in Tehran (herbarium code of PMP756 by Medical Plant Research Center, College of Pharmacy, Tehran Medical University). The seeds were ground in a blender. 1000 grams of the powder was macerated in 4 liters of 70% ethanol. The mixture was gently shaken in the dark at 40°C for 72 hours. The resulting liquid was then filtered and concentrated under vacuum via a rotary evaporator (Stroglass, Italy) at 50°C. The hydroalcoholic seed extract (~150 grams) was stored at 4°C for the treatment of animal groups [33]. The total phenolic content of the extract was determined by the Folin–Ciocalteu colorimetric method described by Singleton and Rossi [35] using gallic acid as a standard. The total flavonoid content of the extracts was determined by colorimetric analysis with aluminum chloride using quercetin as a standard [36].

### 2.2. Animal and Experimental Design

Thirty healthy male Wistar rats (eight-week-old) with an average weight of 240 g were purchased from the Laboratory Animal Center of the Iran University of Medical Sciences. Rats were kept in a well-ventilated place with free access to food and water for one week prior to the experiment. The animal care and experimentation were in accordance with the rules of the Ministry of Health (Code of Ethics: IR.IUMS.FMD.REC.1399.065).

After separating 6 rats as normal control group (NC), hyperglycemia was induced in other rats by the standard method of intraperitoneal injection of streptozotocin (STZ, 55 mg/kg body weight). STZ induces oxidative stress, metabolic changes, and mitochondrial dysfunction in pancreatic cells [37]. A blood sugar level of 250 mg/dL or higher was considered as diabetic [38, 39]. Diabetic rats were randomly placed into four groups: (1) the diabetic control group (DC); (2) group received 100 mg/ml.kg body weight of HESS in normal saline (E-100); (3) group received 200 mg/ml.kg body weight of HESS in normal saline (E-200); (3) group received 300 mg/ml.kg body weight of HESS in normal saline (E-300) [40].

### 2.3. Sampling

Administration of HESS was performed through gavage once a day for 35 days [33]. At the end of the treatment period, the animals were anesthetized by ketamine/xylazine. The blood samples were collected by cardiac puncture and then centrifuged at 2000 g and 23°C for 10 min. The resulting serum was separated and stored at -20°C until biochemical analysis.

Immediately after blood sampling, the pancreas was carefully isolated from the stomach and duodenum. In order to maintain uniformity in the tissue and molecular studies among the groups, three parts were considered in the pancreas [41]. The duodenum side was selected for histopathology, the splenic side for molecular, and the gastric part for gene expression studies. Immediately, the duodenum part was placed in 10% neutral buffered formalin for histological examination, and the splenic and gastric parts were frozen in liquid nitrogen for molecular and biochemical studies, respectively, and then stored at -80°C.

### 2.4. Biochemical Assay

The effects of different doses of HESS on body weight and serum biochemical parameters, including blood glucose and insulin levels, lipid profile, nitric oxide (NO), hs-CRP, TNF*α*, serum oxidants, and antioxidant factors, were evaluated in our previous studies [33, 42].

#### 2.4.1. Tissue Preparation for Oxidant and Antioxidant Tests

The pancreatic tissue was placed in liquid nitrogen in a mortar and frozen, then carefully pounded and crushed into fine powder. In each of the following tests, a certain amount of powdered tissue was homogenized with the lysis buffer containing antiprotease provided by Navand Salamat Company kits (Iran, Naxifer™-Total Oxidant Status Assay Kit (CAT.N.: NS-15016); Naxifer™-Total Antioxidant Capacity Assay Kit (CAT.N.: NS-15012); Nalondi™-Lipid Peroxidation Assay Kit-MDA (CAT.N.: NS-15022)), then centrifuged at 4000 rpm and 4°C for 10 min. The supernatants were used in the following assays.

#### 2.4.2. Total Tissue Antioxidant Capacity (TAC)

Total antioxidant levels in pancreatic tissue were measured as iron-reducing antioxidant capacity (FRAP) [43]. The reduction of Fe^3+^ to Fe^2+^ in the sample was considered as an indicator of antioxidant capacity. In this method, the complex of Fe^2+^ with tripyridyltriazine (Fe^2+^-TPTZ) produces a blue color with a maximum light absorption at 593 nm. Results were compared to a standard curve based on serial dilutions of Fe_2_SO_4_ (range 100-800 *μ*M) in 1 ml FRAP reagent (300 mM acetate buffer, 10 mM TPTZ/HCl solution, and 20 mM ferric chloride). Values are expressed in *μ*mol Fe^2+^/g tissue weight.

#### 2.4.3. Total Tissue Oxidant Status (TOS)

ROS generation was measured by fluorescence analysis using 2,7-dichlorofluorescein diacetate (H_2_DCF-DA) [44]. In this assay, H_2_DCF-DA is deacetylated to H2DCF by endogenous serum esterase and oxidized to dichlorofluorescein (DCF) by types of ROS. DCF fluorescence was measured at 485 nm excitation and 528 nm emission using a Synergy HT microplate reader (BioTek Instruments) set to 37°C. H2DCF is mostly none-fluorescent but becomes highly fluorescent when oxidized to DCF by existent ROS such as superoxide radical anion (O_2_^−^), hydroxyl radical (^•^OH), hydrogen peroxide (H_2_O_2_), hypochlorous acid (HOCl), peroxyl (RO^2-^), alkoxyl (RO.), hydroperoxyl (HO^2-^), and peroxynitrite (ONOO^−^).

#### 2.4.4. Determination of Lipid Peroxidation

The lipid peroxidation (LPO) assay was based on the ability of malondialdehyde (MDA) to conjugate 2-thiobarbituric acid (TBA) to form a pink product with an absorbance maximum at 532 nm [45]. Briefly, 200 *μ*l of each sample was mixed with 20% trichloroacetic acid (TCA), and the resulting precipitate was dispersed in H2SO4 (0.05 M). After thiobarbituric acid (TBA) (0.2% in sodium sulfate) was added, the sample was heated in a boiling water bath for 30 min. Then, the LPO adduct was extracted with n-butanol, and the absorbance was measured at a wavelength of 535 nm. MDA content was expressed as nmol/ml.

#### 2.4.5. Local-RAS and Alternative Route Component Assay

The pancreas is full of digestive enzymes, especially trypsinogen and chymotrypsinogen proteases. If protease inhibitor (such as cocktail (Roche, 04693159001)) is not used, such released and activated proenzymes digest the macromolecules in the tissue homogenate during test. Therefore, neither the activity nor the protein content of considered macromolecules can be measured accurately. Renin, ACE, and ACE2 are also proteases in the pancreas homogenate whose activity decreases in the presence of a protease inhibitor, leading to inaccurate results. Considering oxidative stress as a stimulus for the production of local-RAS components, the measurement of the protein level was considered as a comparison factor. Renin, ACE, and ACE2, as well as Ang II, and Ang-(1-7), were assayed using Rat ELISA Kit (MBS041519, MBS2604372; MBS764117, MBS705139, and MBS2604372, respectively) from MyBioSource Company and multiplate ELISA reader (ELISA Reader-DANA-320, Japan). The tissue value of each parameter was reported based on pg/mg.pro.

### 2.5. Total RNA Extraction, cDNA Synthesis, and Quantitative Real-Time PCR (RT-PCR)

Studies on the gene expression levels of pancreatic cytokines (IL1*β*, IL6, TNF*α*, and IL10) and of angiotensinogen and renin were performed by quantitative real-time PCR (RTqPCR). Approximately, 50 mg of pancreatic tissue was frozen in liquid nitrogen and pulverized in a mortar and pestle with more liquid nitrogen. Powdered tissue was homogenized in TRIzol reagent, and total RNA was extracted using the Total RNA Extraction Kit (ref. FAPDE050, Yektatajhize, Iran) according to the manufacturer's procedure. After quantitative and qualitative evaluation of purified RNA using Nanodrop (ND1000 spectrometer, Yektatajhize, Iran), total RNA was reverse-transcribed into first-strand complementary DNA (cDNA) using the cDNA synthesis (ref. YT4500, Yektatajhize, Iran) according to the manufacturer's instructions. The GAPDH (glyceraldehyde 3-phosphate dehydrogenase) gene was used as an internal control to quantify the expression of target genes. The target genes and the internal control GAPDH were amplified with appropriate primers designed by the NCBI primer designer (Table 1). The synthesized cDNA was quantified by RTqPCR using Super SYBR Green qPCR MasterMix (cat. YT2552, Yektatajhize, Iran) in a 20 *μ*L reaction mixture containing RT-PCR Master Mix (10 *μ*L), forward and reverse primers (1 *μ*L), double distillation of H2O (6 *μ*L), and cDNA (3 *μ*L). Initial denaturation was 95°C for 3 min, 40 cycles of denaturation at 95°C for 15 s, annealing at Tm °C for 30 s, elongation at 72°C for 30 s, and final elongation at 72°C for 5 minutes. The threshold cycle (Ct) of the amplification was determined and normalized by the value of GAPDH. The expression fold change was calculated using the 2^−ΔΔCt^ method [46].

### 2.6. Histopathology

After fixation in 10% neutral buffered formalin, pancreatic tissue was dehydrated with increasing ethanol concentration, clarified in xylene, and embedded in paraffin. The paraffin-embedded tissue was sectioned at 5-6 *μ*m using a rotary microtome (Leica RM2255, Germany); then, the sections were mounted on glass slides. After deparaffinization, the slides were stained with hematoxylin and eosin (H&E). All slides were examined by a veterinary pathologist using an optical microscope equipped with a digital camera under a magnification of ×100 and ×200.

### 2.7. Statistical Analysis

Statistical analysis was performed using SPSS version 22 software and GraphPad Prism 9. The normality of the data was confirmed by the Shapiro-Wilk normality test. Differences between individual groups were determined using one-way ANOVA followed by Tukey's test for pairwise comparisons. Values were expressed as mean ± standard deviation. A value of *P* < 0.05 was considered statistically significant.

## 3. Results

### 3.1. Total Phenols and Flavonoids in HESS

The total content of phenols and flavonoids in the hydroalcoholic extract of *S. securidaca* seeds was 94.05 ± 1.5 mg gallic acid equivalents (GAE)/g (DW) and 46.9 ± 1.7 mg quercetin equivalents (QE)/g (DW), respectively.

### 3.2. Effects of HESS on Insulin and Blood Sugar

As it was mentioned above, the data that support the effects of different doses of HESS on body weight, hs-CRP, lipid profiles, and serum oxidants and antioxidant factors are available through our previous studies in the reference numbers of [33, 42].  Table 2 shows the dose-dependent effects of HESS on insulin and blood sugar. One-way ANOVA showed a significant difference in insulin concentration (*P* < 0.001) and also blood sugar (*P* < 0.001) between groups. Blood insulin concentrations in all three groups treated with HESS were significantly higher than those in the diabetic control group (*P* < 0.05), but the levels were significantly lower than the mean level in the healthy group. Despite the dose-dependently increase in insulin levels among HESS-treated groups, the difference between them was not significant (*P* > 0.05).

The values are mean ± SD. NC: normal control; DC: diabetic control; HESS: hydroalcoholic extract of *S. securidaca* seeds. The groups at the left column have been defined by letters (a–e). Superscript letters (^a–e^) in the middle and right columns denote significant differences (*P* < 0.05) among groups.

### 3.3. Effects of HESS on Oxidative Stress in Pancreas Tissue

**Figure 1:**
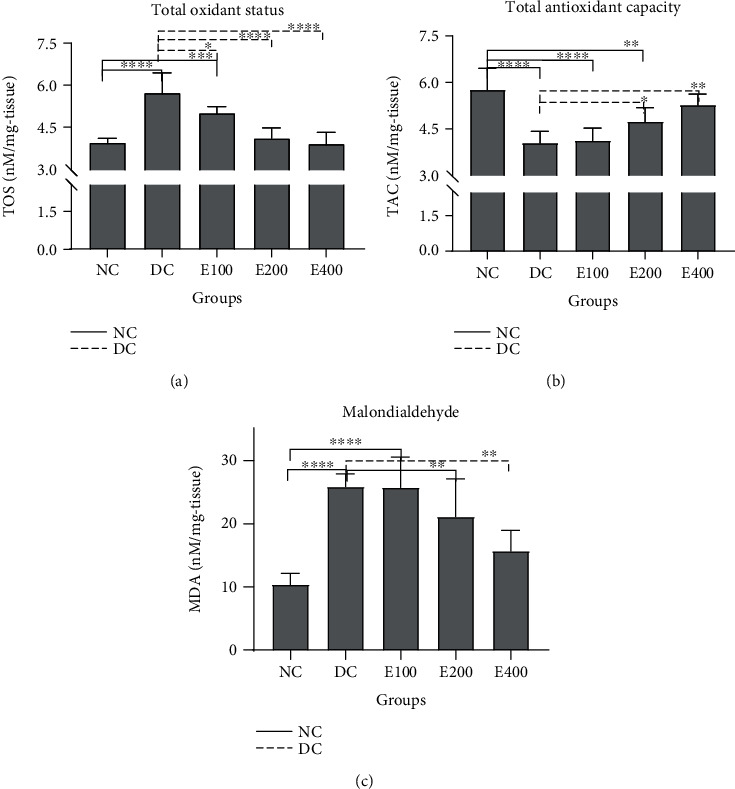
Comparison of oxidative stress and antioxidant levels of pancreatic tissue between the study groups. Values are mean ± SD. (a) Total oxidant status (TOS); (b) Total antioxidant capacity (TAC); (c) Malondialdehyde (MDA). NC: normal control; DC: diabetic control; HESS: hydroalcoholic extract of *S. securidaca* seeds. *P*value =  ^∗^(0.01 − 0.05); ^∗∗^(0.001-0.01); ^∗∗∗^(0.0001-0.001); ^∗∗∗∗^(<0.0001).

Figure 1 compares the levels of oxidative stress markers including total oxidants (TOS) (Figure 1(a)), total antioxidants (TAC) (Figure 1(b)), and MDA (Figure 1(c)) in the pancreatic tissues of the HESS-treated groups with those of healthy and diabetic control groups. As expected, compared to the healthy group, tissue levels of TOS and MDA parameters (*P* < 0.001) were significantly increased in the diabetic control group, whereas TAC levels were significantly decreased in this group (*P* < 0.001). Compared with the diabetic controls, HESS treatment dose-dependently decreased TOS and MDA levels (*P* < 0.001) and increased TAC levels (*P* < 0.001); so that, treatment with the highest dose of HESS altered the aforementioned parameters to the values that statistically had no significant differences from those of healthy controls (*P* > 0.001).

Treatment with the highest HESS dose altered the above parameters to values that were not statistically significantly different from those of healthy controls.

### 3.4. Effects of HESS on the Local-RAS and Alternative Route Components

#### 3.4.1. Local-RAS Components

**Figure 2:**
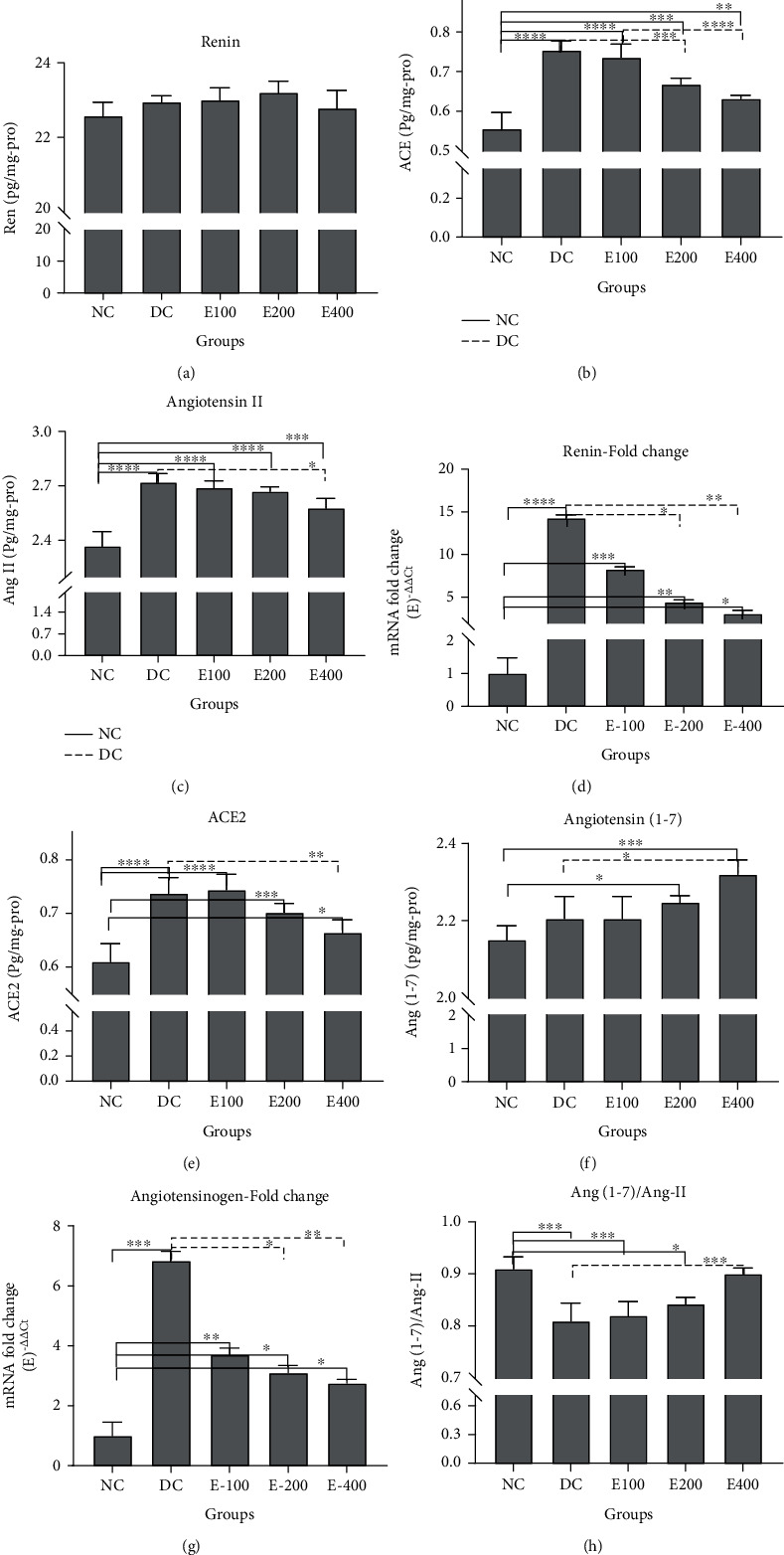
Comparison of pancreatic tissue levels of local-RAS and alternative components among the study groups. Values are mean ± SD. (a) Renin. (b) Angiotensin-converting enzyme (ACE). (c) Angiotensin II (Ang II). (d) Renin mRNA fold changes. (e) Angiotensin-converting enzyme (ACE2). (f) Angiotensin-(1-7) (Ang-(1-7)). (g) Angiotensinogen mRNA fold changes. (h) Tissue ratio of Ang-(1-7)/Ang II. NC: normal control; DC: diabetic control; HESS: hydroalcoholic extract of *S. securidaca* seeds. *P*value =  ^∗^(0.01 − 0.05); ^∗∗^(0.001-0.01); ^∗∗∗^(0.0001-0.001); ^∗∗∗∗^(<0.0001).

According to Figure 2(a), there were no significant differences in renin levels in the tissues of the study groups (*P* > 0.05). Higher levels of ACE and Ang II (Figures 2(b) and 2(c), respectively) were found in the pancreatic tissue of the DC group compared to the NC group (*P* < 0.001). Administration of various doses of HESS did not alter tissue renin levels but dose-dependently decreased ACE and Ang II levels in the tissues of diabetic rats. However, even at the highest dose of HESS, the levels of both parameters were still higher than those in the NC group (*P* < 0.05). Tissue renin levels were expected to be high with increasing ACE and angiotensin levels, but no changes were observed in tissue renin levels. Due to the short half-life of renin (about 10-15 min) [47], this discrepancy was investigated by measuring the expression level of the renin gene. Based on Figure 2(d), it can be seen that the renin gene expression level in the pancreatic tissue of diabetic rats is significantly higher than that of in healthy rats (*P* < 0.05). HESS treatment reduced renin gene expression in a dose-dependent manner in diabetic rats, which became significant at the highest dose of HESS (*P* < 0.05) but remained high compared to the NC group (*P* < 0.05).

#### 3.4.2. Alternative Route Components

Compared with the NC group, the DC group showed a significant increase in ACE2 levels in pancreatic tissue (*P* < 0.001). HESS administration slightly decreased ACE2 levels in diabetic rats in a dose-dependent manner until significant at the highest dose of HESS (*P* < 0.05); however, ACE2 levels were still significantly higher than in the NC group (Figure 2(e)). Tissue levels of Ang-(1-7) were slightly increased in pancreatic tissues of the DC group (*P* > 0.05). HESS treatment dose-dependently increased Ang-(1-7) in diabetic rats, so that it became significant at the highest dose of HESS compared to DC and NC groups (Figure 2(f)).

#### 3.4.3. Compression of Tissue Levels of Ang II and Ang-(1-7)

As shown in Figure 2(g), the expression level of angiotensinogen gene in the pancreatic tissue of the DC group was significantly higher than that of the NC group (*P* < 0.05). HESS therapy dose-dependently decreased geotensinogen gene expression in diabetes, which became significant at the highest dose of HESS compared to the DC group (*P* < 0.05). Angiotensinogen is a precursor for the synthesis of Ang II and Ang-(1-7) synthesis. Thus, it was expected that the decreased levels of Ang II would be associated with increased levels of Ang-(1-7) levels and vice versa. For this, we considered the ratio of two parameters: Ang-(1-7)/Ang II. Ratios greater than 1 indicate an increase in Ang-(1-7), and ratios less than 1 indicate an increase in Ang II and a risk of tissue damage. As shown in Figure 2(h), the Ang-(1-7)/Ang II ratio was less than 1 in all study groups. Ang-(17)/Ang II ratio was significantly higher in the NC group compared to the DC group. HESS treatment increased the ratio in diabetic rats so that the ratio in the HESS400 group was numerically significant compared to the DC group (*P* < 0.05).

### 3.5. Effects of HESS on Proinflammatory and Anti-Inflammatory Cytokines

**Figure 3:**
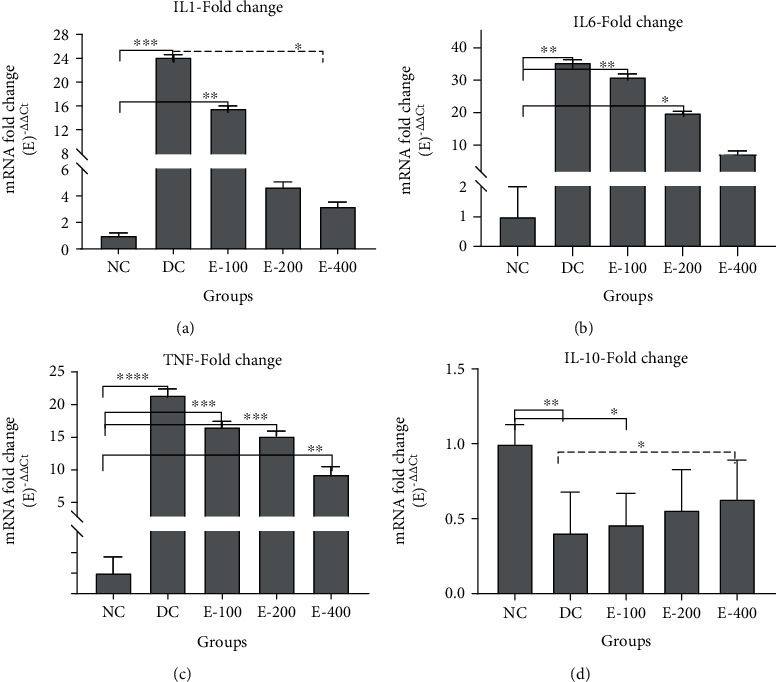
Comparison of mRNA fold changes of proinflammatory (a–c) and anti-inflammatory (d) gene expressions in the pancreatic tissue among the study groups. Values are mean ± SD. NC: normal control; DC: diabetic control; HESS: hydroalcoholic extract of *S. securidaca* seeds). *P*value =  ^∗^(0.01 − 0.05); ^∗∗^(0.001-0.01); ^∗∗∗^(0.0001-0.001); ^∗∗∗∗^(<0.0001).

According to Figure 3, the expression levels of AC, IL1, IL6, and TNF*α* genes were increased in the pancreatic tissue of diabetic rats compared to the healthy group (*P* < 0.05). HESS treatment dose-dependently decreased the gene expression level of these inflammatory factors in diabetic mice, which was significant at the doses of 200 and 400 for IL1 (*P* < 0.05), but the reduction of TNF*α* and IL6 was not significant even at the highest dose of the extract (*P* > 0.05). The IL10 gene expression level in the pancreatic tissue of healthy rats was significantly higher than that of diabetic rats (*P* < 0.05) (Figures 3(d)). HESS therapy increased the expression of the IL-10 gene in diabetic rats, which became significant at the highest dose of HESS compared to DC group (*P* < 0.05).

### 3.6. Effects of HESS on Pancreatic Histology

**Figure 4:**
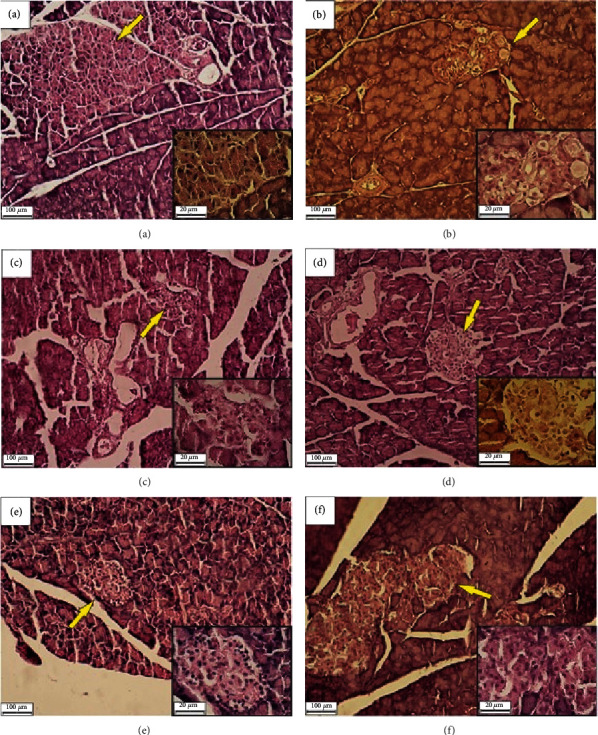
Morphological assessment of pancreatic tissue; H&E staining. Magnification ×100 and ×200. (a) Normal control group with the normal tissue structure. (b) Diabetic control group with an inflamed appearance and Langerhans islets with degraded cells and reduced cell density (c) HESS-100 group: Langerhans islets with degraded cells and reduced cell density. (d) HESS-200 group: Langerhans islets with reduced cell density. (e, f) HESS-400 group: tissue structures are relatively more natural compared to lower HESS-doses but still has a lower cell density than the healthy group. Yellow arrows show islets *of* Langerhans.

Figure 4 compares changes in pancreatic tissue and islets of Langerhans (yellow arrows) among experimental groups.  Figure 4(a) shows a histopathological examination of the pancreas of the NC group with normal tissue and structure. Figures 4(b) and 4(c) show the histopathological complications in the DC and HESS-100 groups with the inflamed appearance of tissue, degenerated islets of Langerhans, and reduced cell density. Treatment with 200-dose of HESS (Figure 4(d)) somewhat reduced the rate of inflammation, although, degraded cells and low cell density still are visible in the islets of Langerhans. Compared to the lower doses of HESS, a relatively healing tissue structure was observed in the HESS-400 group (Figures 4(f) and 4(e)) but still had lower cell density than in the healthy group.

## 4. Discussion

Many herbal remedies such as *S. securidaca* have been used to treat diabetes and are believed to have less toxicity and side effects than current synthetic drugs. However, herbs are full of known and unknown compounds, so it is important to study their effects on the body. Our previous studies demonstrated the antihyperglycemic, antioxidant, and antiatherogenic properties of the hydroalcoholic seed extract of *S. securidaca* [33, 42].

Due to the high metabolic activity of the pancreas in the islets and exocrine parts, the processes of oxidative phosphorylation and oxygen consumption are strongly activated during chronic hyperglycemia [48]. HESS used in the present study dose-dependently attenuated oxidative stress and proinflammatory markers in diabetic pancreas, so that the levels of these markers at treatment with the highest dose of HESS were comparable to those in healthy rats. This improving effect of HESS may be related to the antioxidant protective effect of phenolic and flavonoid compounds on biological systems.

As in previous studies, increased local-RAS activity is associated with hyperglycemia and increased oxidative markers in pancreatic tissue [16, 49–51]. The increase in glucose concentration in beta cells and the subsequent increase in TCA pathway activity, respectively, increase the ATP/ADP ratio, close the ATP-dependent potassium channel, depolarize the membrane, open the calcium channel, increase cytoplasmic calcium, and increase secretion of insulin. In chronic hyperglycemia, continuing the above pathway increases ROS production in beta cells, increases intracellular calcium, and subsequently activates protein kinase C (PKC). PKC increases NADPH oxidase activity, leading to the production of superoxide and other ROS. ROS is an important factor in stimulating several other disruptive pathways including the local-RAS system and generation of local Ang II. On the other hand, Ang II itself is a factor that increases ROS production and oxidative stress, inflammation, and apoptosis. Chu and Leung, using a db/db mouse model, reported that inhibition of the AT1 receptor reduced NADPH oxidase-induced oxidative stress and decreased UCP2 (uncoupling protein 2) expression, which was associated with improved beta-cell insulin secretion and decreased apoptosis-induced beta-cell [20]. In separate studies, Tikellis et al. and Chu et al. reported that hyperglycemia significantly increased the expression of AT1 receptor and angiotensinogen in the diabetic animal model [49, 52]. Zhang et al. in their study on diabetic rat kidney proximal tubular cells reported that hyperglycemia increased angiotensinogen gene expression through generation of ROS, activation of hexosamine biosynthesis, and PKC signaling pathway [53]. Although the roles of members of the mitogen-activated protein kinase (MAPK) family, including p38-MAPK, in inflammation and beta-cell apoptosis have been demonstrated in diabetes [54, 55], their role in Ang II-mediated cellular effects in pancreatic islets needs to be studied. However, studies of vascular smooth muscle cell hypertrophy [56], coronary smooth muscle cells [57], diabetic retinopathy [58], etc. have reported p38MAPK signaling as another stimulus to increase angiotensinogen gene expression. Moderate and high-dose treatment of HESS significantly reduced the expression of the angiotensinogen gene in the pancreas of diabetic rats, but this expression was still higher than that of healthy rats. Previous studies have shown low levels of the antioxidant enzymes superoxide dismutase, glutathione peroxidase, and especially catalase in beta-cells compared with most other tissues, despite being equipped with other antioxidant defenses such as glutaredoxin and thioredoxin.

Angiotensinogen is converted to Ang I by islet-expressed renin. In our study, ELISA showed no significant differences between groups in renin levels, whereas RT-PCR showed a significant increase in renin gene expression in the DC group compared to the NC group. Many studies have reported low levels of renin in various tissues and attributed their results to the effect of plasma renin [47, 59]. This discrepancy in renin results may be related to the half-life of renin, which has been reported in plasma by previous studies [47, 60], but no report of its half-life was found in any tissue. HESS treatment significantly reduced the expression of the renin gene in the pancreas of diabetic rats, but this expression was still higher than that of healthy rats. Previous studies have demonstrated that increased ROS and inflammatory cytokines such as IL-6, IL-1*β*, and TNF*α* through the MAPK/STAT/NF-*κ*B signaling pathway are associated with tissue stimulators of renin gene expression [61, 62]. Therefore, the antioxidant and anti-inflammatory properties of HESS may be the reason for the decrease in renin gene expression by HESS. Therefore, the antioxidant and anti-inflammatory properties of HESS may be responsible for the reduction in renin gene expression by HESS..

Consistent with previous studies, high levels of ACE were detected in the pancreatic tissue from diabetic rats [63], which decreased after treatment with higher doses of HESS, although its levels were still higher than those in healthy rats.

After administration of various doses of HESS, a slow decrease in the concentration of Ang II was observed in diabetic pancreas despite a significant decrease in tissue levels of ACE. High levels of Ang II in the diabetic pancreas may be associated with other production pathways. One of these pathways is the enzymatic catalysis of chymase, a trypsin-like enzyme produced in mast cells. It performs many functions, including an increase in Ang II, MMP9 (matrix of metalloproteinase-9), and TGF-*β*, which are associated with oxidative stress, inflammation, and fibrosis. As a tissue protease whose levels are elevated in metabolic syndrome, chymase is not inhibited by ACE inhibitors and has no effect on blood pressure and plasma renin activity [64, 65].

Two components of the alternative system, ACE2 and Ang-(1-7), are aimed at regulating local-RAS activity and neutralizing the effects of Ang II [66]. There was a significant increase in the tissue level of ACE2 and then Ang-(1-7) with the increase in local-RAS activity in the diabetic control group. HESS had a relatively improved effect on local pancreatic RAS and decreased ACE2 activity but increased tissue Ang-(1-7) unexpectedly. This discrepancy may be due to the activity of other enzymes that partially mimics ACE2 activity. Neprilysin (NEP) is one of these enzymes that can catalyze Ang I to Ang-(1-7). Ang-(1-7) increases insulin sensitivity and *β*-cell function by acting through the Mas receptor [67, 68]. Tissue is affected by the balance between the RAS system and the alternative pathway as assessed by the Ang-(1-7)/Ang II ratio. If a ratio of 1 is considered a balance point, the lower the ratio, the higher the activity of the RAS system and the higher the tissue damage alert. The ratio of Ang-(1-7)/Ang II levels in the pancreatic tissue of diabetic rats was significantly lower than in healthy rats. Treatment with the highest dose of HESS increased this ratio, which was somewhat similar to that of healthy rats.

The main effect of Ang II is mediated by AT1R, whereas Ang-(1-7) interacts only with AT2R and Mas receptor. In the pancreas, AT2R is found only in delta cells, whereas AT1R is found in most pancreatic cells, including *β*-cells. The ACE/Ang II/AT1R pathway is stimulated by oxidative stress and proinflammatory cytokines [68]. On the other hand, high expression of AT1R in the pancreas makes tissues more susceptible to inflammation and fibrosis in pathological conditions due to stimulation of proinflammatory pathways such as NF-*κ*B and expression of the proinflammatory molecules IL-1*β*, IL-6, and TNF*α* [68]. Thus, Ang II, cytokines, and hyperglycemia-induced oxidative stress reinforce each other synergistically. Under normal conditions, the release of proinflammatory cytokines, including TNF*α*, IL-1*β*, and IL-6, is believed to protect the host from changes in homeostasis.

Elevated tissue and serum concentrations of these cytokines in pathological conditions, such as oxidative stress and the development of chronic inflammation, may provide a warning for subsequent complications. For example, high concentration of IL-6 have been reported to be an independent risk factor for type II diabetes and cardiovascular disease [69]. IL-1 together with TNF*α* inhibits insulin synthesis, suppresses insulin-induced secretion, and induces apoptosis in pancreatic *β*-cells, leading to type 1 and type 2 diabetes [68]. As expected, the expression levels of the inflammatory cytokines of IL-1, IL-6, and TNF*α* in the pancreas of diabetic rats were higher than in healthy rats, but the expression levels of the anti-inflammatory cytokine of IL-10 were lower [70–72]. The inhibitory effect of HESS on IL-1 and IL-6 gene expression was more significant than that of TNF*α*. IL-10 can inhibit IL6 and TNF*α* expression and increase the sensitivity of *β*-cells to glucose and insulin secretion. In the study groups, histopathological changes in the pancreas were consistent with histological changes of oxidative factors and inflammatory cytokines. Severe tissue inflammation and decreased cell density were observed in the Langerhans islets of the diabetic control group. Treatment with the HESS was dose-dependently effective in reducing tissue inflammation which was more obvious in the treatment with the highest dose of HESS (400 mg/ml.kg body weight). The histopathological examination of the pancreas and also ameliorative changes in blood insulin and blood sugar via treatment with higher doses of HESS showed the inhibition of oxidative stress, and Ang II level had protective effect on pancreas.

## 5. Conclusions

The highest dose of HESS used in this study had some reducing effect on local-RAS components including angiotensinogen, renin, ACE enzyme, and Ang II, a product of this system. Compared to diabetic control, the highest dose of HESS increased Ang-(1-7) concentration in the alternative pathway without increasing ACE2 levels. Such discrepancies may be due to the involvement of other tissue converting enzymes such as neprilysin, although this needs to be investigated. By reducing local-RAS activity as well as oxidative stress levels and increasing tissue antioxidant levels, decreased tissue levels of proinflammatory interleukins and increased anti-inflammatory interleukins IL10 were observed. Therefore, *S. securidaca* seeds can be considered as a suitable herbal supplement in diabetes treatment protocols.

### 5.1. Suggestions

There are important questions that remain unanswered or at least cannot be answered by animal studies and need cell line studies. For example, is the effect of HESS on pancreatic local-RAS directly through the interaction of phenol-flavonoids with biomolecules or/and through activation of cell signaling? Or is it occurred indirectly, e.g., through free radical scavenging? In addition, previous studies have shown the richness of *S. securidaca* seeds in compounds such as flavonoids, saponins, tannins, and alkaloids. Therefore, more studies should be done to identify the main bioactive components of the seeds of this plant.

## Figures and Tables

**Table 1:** Primer sequences.

Gene	Sequence	PCR product size (bp)	Tm (°C)
IL-1	F: 5′-TTG CCC GTG GAG CTT CCA GG-3′	147	62
	R: 5′-TTC ATC TCG AAG CCT GCA GTG C-3′		
IL-6	F: 5′-TAC CAC TTC ACA AGT CGG AGG C-3′	149	60
	R: 5′-CTG ACA GTG CAT CAT CGC TGT TC-3′		
IL-10	F: 5′-TCC ACT TCC CAG TCA GCC AG-3′	149	62
	R: 5′-TCA CCC AAG TAA CCC TTA AAG TCC-3′		
TNF*α*	F: 5′–TCC CAG ACC CTC ACA CTC AGA TC-3′	159	61
	R: 5′-TCC ACT CCA GCT GCT CCT CTG-3′		
Angiotensinogen	F: 5′-ACT GAG AAG CTA GAG GCT GAG G-3′	168	61
	R: 5′-AAC GAA GAA AGA GAC CAG GGT G-3′		
Renin	F: 5′-TTG AGG AGC GGG GAG TAG AC-3′	168	60
	R: 5′-TCA CTT TGA AGG TCT GGG ATG G-3′		
GAPDH	F: 5′-TCA TCA ACG GCA CAG TCA AGG-3′	154	60
	R: 5′-TTC TGC ATG GTG GTG AAG ACG-3′		

**Table 2:** Comparison of the effects of HESS on insulin and blood sugar in study groups.

Groups	Parameters	
	Insulin (ng/ml)	BS (mg/dl)
NC (a)	1.79 ± 0.14^b^	90.6 ± 5^b^
DC (b)	1.08 ± 0.08^a^	324.6 ± 14^a^
HESS-100 (c)	1.27 ± 0.05^a,b^	221.4 ± 13^a,b,d,e^
HESS-200 (d)	1.4 ± 0.07^a,b^	170.3 ± 8^a,b,c^
HESS-400 (e)	1.42 ± 0.08^a,b^	159.4 ± 14^a,b,c^
ANOVA	*P* < 0.001	*P* < 0.001

## Data Availability

The data presented in this manuscript is available upon request.

## Abbreviations

*S. securidaca*: *Securigera securidaca*
HESS: Hydroalcoholic extract of *S. securidaca* seeds
TCA: Tricarboxylic acid
ACE: Angiotensin-converting enzyme
ACE2: Angiotensin-converting enzyme 2
Ang II: Angiotensin II
Ang-(1-7): Angiotensin (1-7)
NEP: Neprilysin
TOS: Total oxidant status
TAC: Total antioxidant capacity
MDA: Malondialdehyde.
